# 1-Formyl-*r*-2,*c*-6-bis­(4-methoxy­phen­yl)-*t*-3,*t*-5-dimethyl­piperidin-4-one

**DOI:** 10.1107/S1600536809041609

**Published:** 2009-10-23

**Authors:** T. Kavitha, P. Sakthivel, S. Ponnuswamy, S. S. Ilango, M. N. Ponnuswamy

**Affiliations:** a Centre of Advanced Study in Crystallography and Biophysics, University of Madras, Guindy Campus, Chennai 600 025, India; b Department of Chemistry, Government Arts College (Autonomous), Coimbatore 641 018, Tamil Nadu, India

## Abstract

In the title compound, C_22_H_25_NO_4_, the piperidine ring adopts a distorted boat conformation. The methyl groups at the 3 and 5 positions of the piperidine ring are in axial and equatorial orientations, respectively. Both H and O atoms in the aldehyde group are disordered over two positions with occupancies of 0.534 (5) and 0.466 (5). In the crystal, the mol­ecules are linked into a three-dimensional network by C—H⋯O hydrogen bonds.

## Related literature

For general background to piperidine derivatives, see: Escolano & Amat (2006 ▶); Wang & Wuorola (1992 ▶); Grishina *et al.* (1994 ▶). For hydrogen-bond motifs, see: Bernstein *et al.* (1995 ▶).
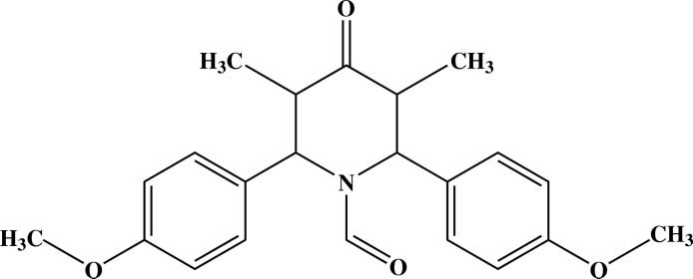


## Experimental

### 

#### Crystal data

C_22_H_25_NO_4_
*M* *_r_* = 367.43
Monoclinic, 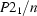

*a* = 11.0954 (4) Å
*b* = 14.5407 (3) Å
*c* = 12.7050 (4) Å
β = 110.977 (1)°
*V* = 1913.91 (10) Å^3^
*Z* = 4
Mo *K*α radiation
μ = 0.09 mm^−1^
*T* = 293 K
0.30 × 0.20 × 0.20 mm

#### Data collection

Bruker Kappa APEXII CCD area-detector diffractometer
Absorption correction: multi-scan (*SADABS*; Sheldrick, 2001 ▶) *T*_min_ = 0.974, *T*_max_ = 0.974
24720 measured reflections
5524 independent reflections
3703 reflections with *I* > 2σ(*I*)
*R*_int_ = 0.029

#### Refinement

*R*[*F* ^2^ > 2σ(*F* ^2^)] = 0.048
*wR*(*F* ^2^) = 0.140
*S* = 1.05
5524 reflections
256 parameters
H-atom parameters constrained
Δρ_max_ = 0.23 e Å^−3^
Δρ_min_ = −0.23 e Å^−3^

### 

Data collection: *APEX2* (Bruker, 2004 ▶); cell refinement: *SAINT* (Bruker, 2004 ▶); data reduction: *SAINT*; program(s) used to solve structure: *SHELXS97* (Sheldrick, 2008 ▶); program(s) used to refine structure: *SHELXL97* (Sheldrick, 2008 ▶); molecular graphics: *ORTEP-3* (Farrugia, 1997 ▶); software used to prepare material for publication: *SHELXL97* and *PLATON* (Spek, 2009 ▶).

## Supplementary Material

Crystal structure: contains datablocks I, global. DOI: 10.1107/S1600536809041609/ci2909sup1.cif

Structure factors: contains datablocks I. DOI: 10.1107/S1600536809041609/ci2909Isup2.hkl

Additional supplementary materials:  crystallographic information; 3D view; checkCIF report

## Figures and Tables

**Table 1:** Hydrogen-bond geometry (Å, °)

*D*—H⋯*A*	*D*—H	H⋯*A*	*D*⋯*A*	*D*—H⋯*A*
C3—H3⋯O1^i^	0.98	2.48	3.429 (2)	163
C15—H15*C*⋯O2^ii^	0.96	2.53	3.240 (4)	131
C20—H20⋯O1^iii^	0.93	2.51	3.432 (2)	171

Symmetry codes: (i) 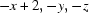; (ii) 
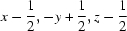
; (iii) 
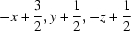
.

**Fig. 1.**
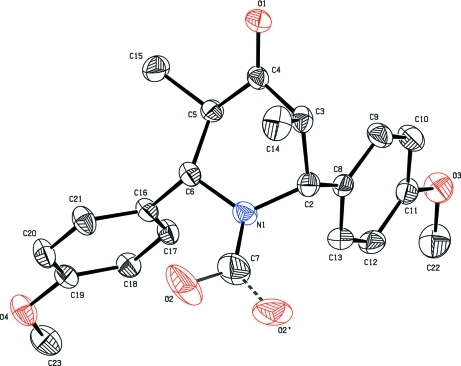
The molecular structure of the title compound. Displacement ellipsoids are drawn at the 30% probability level. Both disorder components are shown. H atoms have been omitted for clarity.

**Fig. 2.**
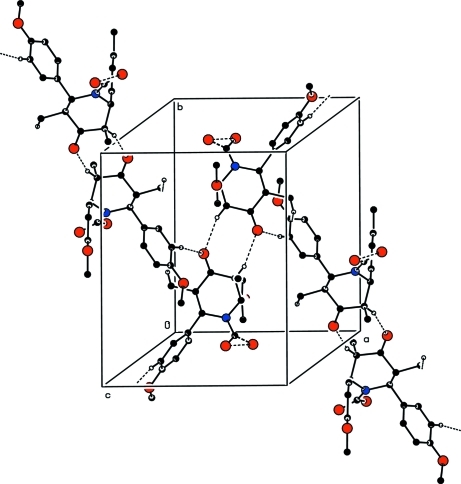
The crystal packing of the title compound. H atoms not involved in hydrogen bonding (dashed lines) have been omitted.

## supplementary crystallographic information

### Comment

The piperidine ring is a common feature occurring in many biologically active natural products and therapeutic agents. Piperidine containing entities constitute important targets for pharmaceutical research (Escolano & Amat, 2006). Piperidine derivatives, namely 4-piperidones are synthetic intermediates in the preparation of various alkaloids and pharmaceutical products (Wang *et al.*, 1992; Grishina *et al.*, 1994).

In the title molecule (Fig.1), the piperidine ring adopts a distorted boat conformation. The methyl groups at 3 and 5 positions of the piperidine ring are in axial and equatorial orientations [N1—C2—C3—C14 = -63.02 (15)° and N1—C6—C5—C15 = 176.60 (11)°]. The phenyl rings at 2 and 6 positions of the piperidine ring are axially [C4—C3—C2—C8 = -68.32 (14)°] and equatorially [C4—C5—C6—C16 = 176.69 (10)°] oriented. The dihedral angle between the two phenyl rings is 41.97 (8)°.

Centrosymmetrically related molecules form *R*_2_^2^(8) (Bernstein *et al.*, 1995) dimers through C—H···O hydrogen bonds involving atoms C3 and O1. The dimers are linked into a zigzag C(8) chain running along the *b* axis by intermolecular C—H···O hydrogen bonds involving atoms C20 and O1 (Table 1). Further, C15—H15C···O2 interactions link the chains along the *c* axis to form a three-dimensional network.

### Experimental

An ice-cold solution of acetic-formic anhydride was prepared from acetic anhydride (10 ml) and 85% formic acid (5 ml) and was added slowly to a cold solution of r-2,c-6-bis(4-methoxyphenyl)-t-3,t-5-dimethylpiperidin-4-one (1.69 g) in benzene (30 ml). The reaction mixture was stirred at room temperature for 5 h. The organic layer was separated, dried over anhydrous Na_2_SO_4_ and concentrated. The resulting mass was purified by crystallization from benzene-petroleum ether (333–353 K) in the ratio 1:1.

### Refinement

The O and H atoms of the formyl group is disordered over two positions with occupancies of 0.534 (5) and 0.466 (5). H atoms were positioned geometrically (C-H = 0.93–0.98 Å) and allowed to ride on their parent atoms, with *U*_iso_(H) = 1.5*U*_eq_(C_methyl_) and 1.2*U*_eq_(C). A rotating group model was used for the methoxy methyl groups.

### Crystal data

**Table:**

C_22_H_25_NO_4_	*F*(000) = 784
*M**_r_* = 367.43	*D*_x_ = 1.275 Mg m^−^^3^
Monoclinic, *P*2_1_/*n*	Mo *K*α radiation, λ = 0.71073 Å
Hall symbol: -P 2yn	Cell parameters from 5524 reflections
*a* = 11.0954 (4) Å	θ = 2.1–29.9°
*b* = 14.5407 (3) Å	µ = 0.09 mm^−^^1^
*c* = 12.7050 (4) Å	*T* = 293 K
β = 110.977 (1)°	Block, colourless
*V* = 1913.91 (10) Å^3^	0.30 × 0.20 × 0.20 mm
*Z* = 4	

### Data collection

**Table:**

Bruker Kappa APEXII CCD area-detector diffractometer	5524 independent reflections
Radiation source: fine-focus sealed tube	3703 reflections with *I* > 2σ(*I*)
graphite	*R*_int_ = 0.029
ω and φ scans	θ_max_ = 29.9°, θ_min_ = 2.1°
Absorption correction: multi-scan (*SADABS*; Sheldrick, 2001)	*h* = −15→12
*T*_min_ = 0.974, *T*_max_ = 0.974	*k* = −20→19
24720 measured reflections	*l* = −17→17

### Refinement

**Table:**

Refinement on *F*^2^	Primary atom site location: structure-invariant direct methods
Least-squares matrix: full	Secondary atom site location: difference Fourier map
*R*[*F*^2^ > 2σ(*F*^2^)] = 0.048	Hydrogen site location: inferred from neighbouring sites
*wR*(*F*^2^) = 0.140	H-atom parameters constrained
*S* = 1.05	*w* = 1/[σ^2^(*F*_o_^2^) + (0.0598*P*)^2^ + 0.3189*P*] where *P* = (*F*_o_^2^ + 2*F*_c_^2^)/3
5524 reflections	(Δ/σ)_max_ = 0.001
256 parameters	Δρ_max_ = 0.23 e Å^−^^3^
0 restraints	Δρ_min_ = −0.23 e Å^−^^3^

### Special details

**Table:**

Geometry. All e.s.d.'s (except the e.s.d. in the dihedral angle between two l.s. planes) are estimated using the full covariance matrix. The cell e.s.d.'s are taken into account individually in the estimation of e.s.d.'s in distances, angles and torsion angles; correlations between e.s.d.'s in cell parameters are only used when they are defined by crystal symmetry. An approximate (isotropic) treatment of cell e.s.d.'s is used for estimating e.s.d.'s involving l.s. planes.
Refinement. Refinement of *F*^2^ against ALL reflections. The weighted *R*-factor *wR* and goodness of fit *S* are based on *F*^2^, conventional *R*-factors *R* are based on *F*, with *F* set to zero for negative *F*^2^. The threshold expression of *F*^2^ > σ(*F*^2^) is used only for calculating *R*-factors(gt) *etc*. and is not relevant to the choice of reflections for refinement. *R*-factors based on *F*^2^ are statistically about twice as large as those based on *F*, and *R*- factors based on ALL data will be even larger.

### Fractional atomic coordinates and isotropic or equivalent isotropic displacement parameters (Å^2^)

**Table:**

	*x*	*y*	*z*	*U*_iso_*/*U*_eq_	Occ. (<1)
O1	0.87856 (11)	0.03534 (7)	0.05971 (10)	0.0574 (3)	
O2	1.0929 (3)	0.3733 (2)	0.3086 (2)	0.1143 (15)	0.534 (5)
O2'	1.2050 (3)	0.3881 (2)	0.2198 (3)	0.1036 (16)	0.466 (5)
O3	0.92438 (12)	0.33025 (8)	−0.37580 (9)	0.0647 (3)	
O4	0.60958 (11)	0.58982 (7)	0.13202 (9)	0.0600 (3)	
N1	1.03399 (11)	0.28962 (8)	0.15129 (9)	0.0446 (3)	
C2	1.08936 (13)	0.22990 (9)	0.08641 (11)	0.0433 (3)	
H2	1.1831	0.2389	0.1177	0.052*	
C3	1.06451 (13)	0.13017 (10)	0.10905 (12)	0.0455 (3)	
H3	1.0976	0.0906	0.0631	0.055*	
C4	0.92172 (13)	0.11238 (9)	0.07622 (11)	0.0408 (3)	
C5	0.83662 (12)	0.19522 (9)	0.06811 (11)	0.0397 (3)	
H5	0.8182	0.2227	−0.0064	0.048*	
C6	0.90568 (13)	0.26797 (9)	0.15605 (10)	0.0402 (3)	
H6	0.9198	0.2415	0.2305	0.048*	
C7	1.1102 (2)	0.34871 (18)	0.22651 (19)	0.0930 (7)	
H7	1.1815	0.3720	0.2133	0.112*	0.534 (5)
H7'	1.0887	0.3619	0.2918	0.112*	0.466 (5)
C8	1.04508 (12)	0.25691 (9)	−0.03708 (11)	0.0405 (3)	
C9	1.01446 (16)	0.19282 (10)	−0.12353 (13)	0.0536 (4)	
H9	1.0200	0.1305	−0.1062	0.064*	
C10	0.97608 (17)	0.21971 (11)	−0.23422 (13)	0.0582 (4)	
H10	0.9554	0.1753	−0.2906	0.070*	
C11	0.96764 (14)	0.31160 (10)	−0.26313 (12)	0.0472 (3)	
C12	1.00016 (15)	0.37652 (10)	−0.17907 (13)	0.0517 (4)	
H12	0.9968	0.4387	−0.1968	0.062*	
C13	1.03795 (14)	0.34869 (10)	−0.06774 (13)	0.0496 (3)	
H13	1.0593	0.3932	−0.0115	0.060*	
C14	1.13194 (17)	0.10344 (14)	0.23312 (14)	0.0675 (5)	
H14A	1.2219	0.1187	0.2568	0.101*	
H14B	1.1227	0.0385	0.2417	0.101*	
H14C	1.0934	0.1364	0.2786	0.101*	
C15	0.70806 (15)	0.16822 (11)	0.07688 (17)	0.0619 (4)	
H15A	0.6531	0.2213	0.0637	0.093*	
H15B	0.7224	0.1444	0.1509	0.093*	
H15C	0.6675	0.1219	0.0216	0.093*	
C16	0.82499 (13)	0.35393 (9)	0.14503 (11)	0.0406 (3)	
C17	0.79081 (14)	0.40931 (9)	0.05079 (11)	0.0435 (3)	
H17	0.8174	0.3932	−0.0084	0.052*	
C18	0.71800 (14)	0.48820 (9)	0.04184 (11)	0.0444 (3)	
H18	0.6960	0.5246	−0.0225	0.053*	
C19	0.67825 (13)	0.51235 (9)	0.12957 (11)	0.0433 (3)	
C20	0.70878 (16)	0.45640 (11)	0.22319 (12)	0.0533 (4)	
H20	0.6803	0.4715	0.2815	0.064*	
C21	0.78103 (16)	0.37843 (10)	0.23047 (12)	0.0518 (4)	
H21	0.8009	0.3413	0.2940	0.062*	
C22	0.9119 (2)	0.42401 (13)	−0.40951 (16)	0.0748 (5)	
H22A	0.8799	0.4276	−0.4903	0.112*	
H22B	0.9946	0.4535	−0.3797	0.112*	
H22C	0.8527	0.4543	−0.3813	0.112*	
C23	0.58744 (18)	0.65403 (12)	0.04332 (15)	0.0655 (5)	
H23A	0.5442	0.7070	0.0578	0.098*	
H23B	0.5346	0.6263	−0.0265	0.098*	
H23C	0.6685	0.6723	0.0386	0.098*	

### Atomic displacement parameters (Å^2^)

**Table:**

	*U*^11^	*U*^22^	*U*^33^	*U*^12^	*U*^13^	*U*^23^
O1	0.0733 (7)	0.0333 (5)	0.0769 (7)	−0.0017 (5)	0.0407 (6)	−0.0034 (5)
O2	0.096 (2)	0.140 (3)	0.085 (2)	0.0135 (19)	0.0052 (15)	−0.072 (2)
O2'	0.0510 (18)	0.085 (2)	0.156 (3)	−0.0229 (15)	0.0140 (18)	−0.046 (2)
O3	0.0798 (8)	0.0614 (7)	0.0550 (6)	0.0062 (6)	0.0265 (6)	0.0104 (5)
O4	0.0746 (7)	0.0486 (6)	0.0589 (6)	0.0254 (5)	0.0266 (5)	0.0045 (5)
N1	0.0411 (6)	0.0452 (6)	0.0437 (6)	−0.0008 (5)	0.0105 (5)	−0.0090 (5)
C2	0.0353 (6)	0.0446 (8)	0.0507 (7)	0.0034 (5)	0.0162 (6)	−0.0003 (6)
C3	0.0479 (8)	0.0426 (8)	0.0514 (8)	0.0141 (6)	0.0243 (6)	0.0085 (6)
C4	0.0526 (8)	0.0337 (7)	0.0423 (7)	0.0041 (6)	0.0244 (6)	0.0024 (5)
C5	0.0399 (6)	0.0335 (6)	0.0467 (7)	0.0032 (5)	0.0166 (5)	0.0013 (5)
C6	0.0479 (7)	0.0379 (7)	0.0366 (6)	0.0073 (6)	0.0173 (5)	0.0014 (5)
C7	0.0728 (13)	0.1154 (18)	0.0846 (14)	−0.0275 (13)	0.0205 (11)	−0.0533 (13)
C8	0.0373 (6)	0.0380 (7)	0.0518 (7)	0.0001 (5)	0.0227 (6)	0.0001 (6)
C9	0.0768 (11)	0.0344 (7)	0.0575 (9)	0.0039 (7)	0.0337 (8)	0.0006 (6)
C10	0.0841 (12)	0.0440 (8)	0.0535 (9)	0.0016 (8)	0.0330 (8)	−0.0051 (7)
C11	0.0452 (7)	0.0494 (8)	0.0525 (8)	0.0033 (6)	0.0242 (6)	0.0044 (6)
C12	0.0569 (9)	0.0368 (7)	0.0652 (9)	−0.0021 (6)	0.0264 (7)	0.0065 (7)
C13	0.0550 (8)	0.0379 (7)	0.0588 (8)	−0.0072 (6)	0.0237 (7)	−0.0043 (6)
C14	0.0601 (10)	0.0778 (12)	0.0615 (10)	0.0226 (9)	0.0179 (8)	0.0232 (9)
C15	0.0469 (8)	0.0496 (9)	0.0950 (13)	0.0020 (7)	0.0323 (8)	−0.0004 (8)
C16	0.0500 (7)	0.0346 (7)	0.0385 (6)	0.0058 (5)	0.0175 (5)	−0.0017 (5)
C17	0.0535 (8)	0.0428 (7)	0.0378 (6)	0.0063 (6)	0.0208 (6)	−0.0009 (5)
C18	0.0526 (8)	0.0398 (7)	0.0402 (7)	0.0050 (6)	0.0159 (6)	0.0042 (6)
C19	0.0473 (7)	0.0361 (7)	0.0460 (7)	0.0059 (6)	0.0160 (6)	−0.0032 (5)
C20	0.0732 (10)	0.0496 (9)	0.0462 (8)	0.0149 (7)	0.0324 (7)	0.0006 (6)
C21	0.0746 (10)	0.0454 (8)	0.0412 (7)	0.0165 (7)	0.0280 (7)	0.0066 (6)
C22	0.0856 (13)	0.0710 (12)	0.0707 (11)	0.0147 (10)	0.0316 (10)	0.0259 (9)
C23	0.0765 (11)	0.0466 (9)	0.0673 (10)	0.0210 (8)	0.0183 (9)	0.0075 (8)

### Geometric parameters (Å, °)

**Table:**

O1—C4	1.2067 (16)	C10—C11	1.380 (2)
O2—C7	1.182 (3)	C10—H10	0.93
O2'—C7	1.227 (4)	C11—C12	1.373 (2)
O3—C11	1.3642 (18)	C12—C13	1.384 (2)
O3—C22	1.421 (2)	C12—H12	0.93
O4—C19	1.3665 (16)	C13—H13	0.93
O4—C23	1.416 (2)	C14—H14A	0.96
N1—C7	1.337 (2)	C14—H14B	0.96
N1—C2	1.4743 (17)	C14—H14C	0.96
N1—C6	1.4803 (17)	C15—H15A	0.96
C2—C8	1.5183 (19)	C15—H15B	0.96
C2—C3	1.523 (2)	C15—H15C	0.96
C2—H2	0.98	C16—C17	1.3787 (18)
C3—C4	1.509 (2)	C16—C21	1.3865 (18)
C3—C14	1.533 (2)	C17—C18	1.3840 (19)
C3—H3	0.98	C17—H17	0.93
C4—C5	1.5111 (18)	C18—C19	1.3829 (19)
C5—C15	1.521 (2)	C18—H18	0.93
C5—C6	1.5302 (18)	C19—C20	1.379 (2)
C5—H5	0.98	C20—C21	1.372 (2)
C6—C16	1.5145 (18)	C20—H20	0.93
C6—H6	0.98	C21—H21	0.93
C7—H7	0.93	C22—H22A	0.96
C7—H7'	0.96	C22—H22B	0.96
C8—C13	1.385 (2)	C22—H22C	0.96
C8—C9	1.387 (2)	C23—H23A	0.96
C9—C10	1.372 (2)	C23—H23B	0.96
C9—H9	0.93	C23—H23C	0.96
			
C11—O3—C22	117.82 (13)	C12—C11—C10	118.99 (14)
C19—O4—C23	117.70 (12)	C11—C12—C13	119.53 (14)
C7—N1—C2	119.66 (14)	C11—C12—H12	120.2
C7—N1—C6	118.59 (14)	C13—C12—H12	120.2
C2—N1—C6	119.80 (10)	C12—C13—C8	122.36 (14)
N1—C2—C8	112.33 (11)	C12—C13—H13	118.8
N1—C2—C3	108.39 (11)	C8—C13—H13	118.8
C8—C2—C3	115.34 (12)	C3—C14—H14A	109.5
N1—C2—H2	106.8	C3—C14—H14B	109.5
C8—C2—H2	106.8	H14A—C14—H14B	109.5
C3—C2—H2	106.8	C3—C14—H14C	109.5
C4—C3—C2	110.75 (11)	H14A—C14—H14C	109.5
C4—C3—C14	108.56 (12)	H14B—C14—H14C	109.5
C2—C3—C14	112.39 (13)	C5—C15—H15A	109.5
C4—C3—H3	108.3	C5—C15—H15B	109.5
C2—C3—H3	108.3	H15A—C15—H15B	109.5
C14—C3—H3	108.3	C5—C15—H15C	109.5
O1—C4—C3	121.28 (12)	H15A—C15—H15C	109.5
O1—C4—C5	121.92 (13)	H15B—C15—H15C	109.5
C3—C4—C5	116.79 (11)	C17—C16—C21	117.74 (12)
C4—C5—C15	111.63 (11)	C17—C16—C6	122.14 (11)
C4—C5—C6	111.35 (11)	C21—C16—C6	120.11 (12)
C15—C5—C6	111.25 (12)	C16—C17—C18	121.77 (12)
C4—C5—H5	107.5	C16—C17—H17	119.1
C15—C5—H5	107.5	C18—C17—H17	119.1
C6—C5—H5	107.5	C19—C18—C17	119.31 (12)
N1—C6—C16	111.47 (11)	C19—C18—H18	120.3
N1—C6—C5	110.78 (10)	C17—C18—H18	120.3
C16—C6—C5	112.22 (10)	O4—C19—C20	115.68 (12)
N1—C6—H6	107.4	O4—C19—C18	124.68 (12)
C16—C6—H6	107.4	C20—C19—C18	119.63 (12)
C5—C6—H6	107.4	C21—C20—C19	120.17 (12)
O2—C7—O2'	109.5 (3)	C21—C20—H20	119.9
O2—C7—N1	124.4 (3)	C19—C20—H20	119.9
O2'—C7—N1	126.1 (3)	C20—C21—C16	121.34 (13)
O2—C7—H7	117.8	C20—C21—H21	119.3
N1—C7—H7	117.8	C16—C21—H21	119.3
O2'—C7—H7'	116.8	O3—C22—H22A	109.5
N1—C7—H7'	117.2	O3—C22—H22B	109.5
C13—C8—C9	116.86 (13)	H22A—C22—H22B	109.5
C13—C8—C2	120.31 (12)	O3—C22—H22C	109.5
C9—C8—C2	122.79 (12)	H22A—C22—H22C	109.5
C10—C9—C8	121.23 (14)	H22B—C22—H22C	109.5
C10—C9—H9	119.4	O4—C23—H23A	109.5
C8—C9—H9	119.4	O4—C23—H23B	109.5
C9—C10—C11	121.01 (14)	H23A—C23—H23B	109.5
C9—C10—H10	119.5	O4—C23—H23C	109.5
C11—C10—H10	119.5	H23A—C23—H23C	109.5
O3—C11—C12	125.11 (13)	H23B—C23—H23C	109.5
O3—C11—C10	115.89 (13)		
			
C7—N1—C2—C8	−109.80 (19)	N1—C2—C8—C9	−140.48 (13)
C6—N1—C2—C8	86.30 (14)	C3—C2—C8—C9	−15.60 (18)
C7—N1—C2—C3	121.58 (18)	C13—C8—C9—C10	−1.5 (2)
C6—N1—C2—C3	−42.33 (16)	C2—C8—C9—C10	−179.28 (14)
N1—C2—C3—C4	58.59 (14)	C8—C9—C10—C11	0.5 (3)
C8—C2—C3—C4	−68.32 (14)	C22—O3—C11—C12	0.1 (2)
N1—C2—C3—C14	−63.02 (15)	C22—O3—C11—C10	178.86 (15)
C8—C2—C3—C14	170.07 (11)	C9—C10—C11—O3	−177.91 (14)
C2—C3—C4—O1	160.79 (13)	C9—C10—C11—C12	0.9 (2)
C14—C3—C4—O1	−75.37 (17)	O3—C11—C12—C13	177.39 (14)
C2—C3—C4—C5	−20.74 (16)	C10—C11—C12—C13	−1.3 (2)
C14—C3—C4—C5	103.09 (14)	C11—C12—C13—C8	0.3 (2)
O1—C4—C5—C15	19.05 (19)	C9—C8—C13—C12	1.1 (2)
C3—C4—C5—C15	−159.40 (12)	C2—C8—C13—C12	178.93 (13)
O1—C4—C5—C6	144.05 (13)	N1—C6—C16—C17	60.77 (17)
C3—C4—C5—C6	−34.40 (15)	C5—C6—C16—C17	−64.16 (17)
C7—N1—C6—C16	57.6 (2)	N1—C6—C16—C21	−120.35 (14)
C2—N1—C6—C16	−138.34 (12)	C5—C6—C16—C21	114.73 (15)
C7—N1—C6—C5	−176.69 (17)	C21—C16—C17—C18	1.7 (2)
C2—N1—C6—C5	−12.62 (16)	C6—C16—C17—C18	−179.42 (13)
C4—C5—C6—N1	51.38 (14)	C16—C17—C18—C19	0.0 (2)
C15—C5—C6—N1	176.60 (11)	C23—O4—C19—C20	173.65 (15)
C4—C5—C6—C16	176.69 (10)	C23—O4—C19—C18	−5.9 (2)
C15—C5—C6—C16	−58.09 (15)	C17—C18—C19—O4	177.91 (13)
C2—N1—C7—O2	−150.5 (3)	C17—C18—C19—C20	−1.7 (2)
C6—N1—C7—O2	13.6 (4)	O4—C19—C20—C21	−177.95 (15)
C2—N1—C7—O2'	32.2 (4)	C18—C19—C20—C21	1.7 (2)
C6—N1—C7—O2'	−163.7 (3)	C19—C20—C21—C16	0.0 (3)
N1—C2—C8—C13	41.78 (17)	C17—C16—C21—C20	−1.7 (2)
C3—C2—C8—C13	166.66 (12)	C6—C16—C21—C20	179.39 (14)

### Hydrogen-bond geometry (Å, °)

**Table:**

*D*—H···*A*	*D*—H	H···*A*	*D*···*A*	*D*—H···*A*
C3—H3···O1^i^	0.98	2.48	3.429 (2)	163
C15—H15C···O2^ii^	0.96	2.53	3.240 (4)	131
C20—H20···O1^iii^	0.93	2.51	3.432 (2)	171

Symmetry codes: (i) −*x*+2, −*y*, −*z*; (ii) *x*−1/2, −*y*+1/2, *z*−1/2; (iii) −*x*+3/2, *y*+1/2, −*z*+1/2.
